# Rupture of the anterolateral papillary muscle without significant coronary stenosis: a surgical emergency

**DOI:** 10.1093/ehjcr/ytaf337

**Published:** 2025-07-17

**Authors:** Débora Sá, Francisco Sousa, João Adriano Sousa, João Manuel Rodrigues

**Affiliations:** Cardiology Department, Hospital Central do Funchal, Avenida Luís de Camões n° 57 Funchal, Madeira 9004-514, Portugal; Cardiology Department, Hospital Central do Funchal, Avenida Luís de Camões n° 57 Funchal, Madeira 9004-514, Portugal; Cardiology Department, Hospital Central do Funchal, Avenida Luís de Camões n° 57 Funchal, Madeira 9004-514, Portugal; Cardiothoraric Department, Hospital Central do Funchal, Avenida Luís de Camões n° 57 Funchal, Madeira 9004-514, Portugal

## Case description

**Figure 1:**
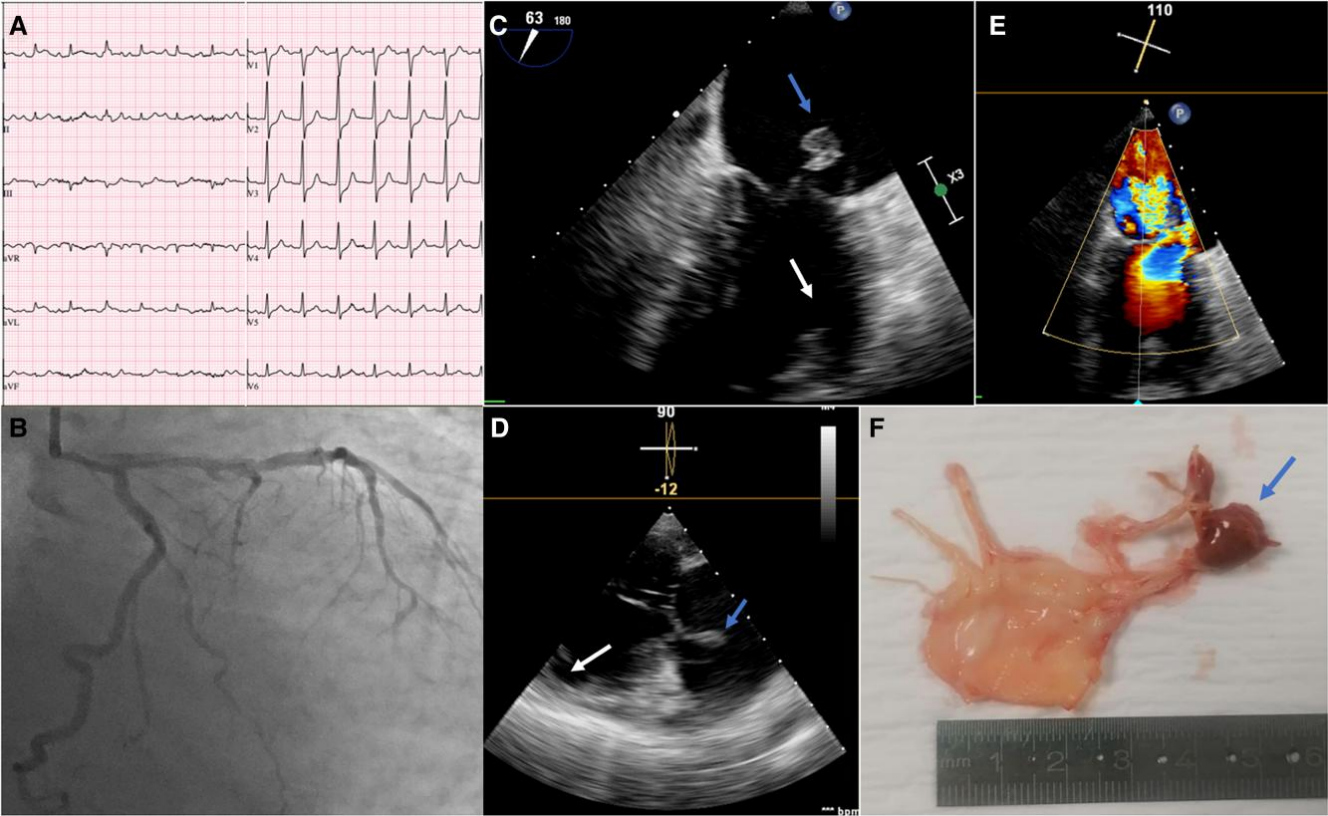
(*A*) Electrocardiogram showing ST-segment depression in the anterior leads. (*B*) Coronary angiography revealing no significant lesions in the left coronary circulation. (*C*, *D*) Transoesophageal echocardiography images demonstrating a mass attached to the mitral valve, prolapsing into the left atrium during systole and causing severe mitral regurgitation, as shown in colour Doppler imaging (*E*). (*F*) Surgical specimens of the ruptured anterolateral papillary muscle (APM) and mitral leaflets. Blue arrow: head of the APM attached to the mitral valve; white arrow: disruption zone.

Seventy-seven-year-old male with a history of hypertension and dyslipidaemia presented to the emergency department with 2-day onset chest pain and dyspnoea, arriving in cardiogenic shock. Electrocardiogram (ECG) showed ST-segment depression in the anterior leads (*Figure 1A*). Coronary angiography revealed no significant lesions, only slow-flow in the left anterior descending (LAD) artery, which improved afterward (*Figure 1B*).

Bedside echocardiography showed posterior leaflet prolapse with acute severe mitral regurgitation. The left ventricle (LV) was not dilated, with mildly reduced systolic function (LVEF 44%; LVEDD 46 mm; LVESD 31 mm; septum/posterior wall 10/9 mm) and akinesia of the mid-apical anterior, anterolateral, and inferolateral segments. The left atrium (LA) was mildly enlarged (diameter 45 mm; volume 72 mL, 42 mL/m²). Transoesophageal echocardiography confirmed severe MR due to rupture of the anterolateral papillary muscle (APM), prolapsing into the LA (*Figure 1C–E*; Blue arrow—head of APM attached to the mitral valve; White arrow—disruption zone). Cardiac biomarkers were markedly elevated.

The patient’s condition continued to deteriorate despite intra-aortic balloon pump support and aminergic therapy, necessitating emergent surgery. A biological prosthesis was successfully implanted. Surgical specimens of the ruptured APM and mitral leaflets are shown in *Figure 1F*.

Following surgery, the patient improved hemodynamically, emerging from shock. However, prolonged ventilator weaning required a tracheostomy, and multiple infections ensued, ultimately leading to death on day 62 of hospitalization.

The main differential diagnoses include a myocardial infarction with non-obstructive coronary arteries (MINOCA) and rupture secondary to degenerative mitral valve prolapse (MVP). Given the troponin elevation and regional wall motion abnormalities, ischaemia was considered. The absence of significant coronary lesions on angiography led us to hypothesize a transient reduction in perfusion—possibly due to a spontaneously reperfused thrombotic occlusion, prolonged vasospasm, or microvascular dysfunction—affecting the blood supply of the APM (usually LAD and LCx).^1^ However, rupture as a complication of pre-existing MVP remains a plausible alternative. In this context, mid-apical wall motion abnormalities may reflect chronic mechanical stress and fibrosis rather than acute ischaemia,^2^ and troponin elevation may occur in the setting of acute severe mitral regurgitation. However, echocardiography revealed no significant dilatation of LA or LV that would support this diagnosis. Also, no prior echocardiographic studies were available to assess for underlying mitral valve disease.

This case is limited by the absence of histological examination of the excised papillary muscle, as well as the lack of cardiac magnetic resonance and intracoronary imaging, which could have helped clarify the underlying mechanism.

Regardless of the underlying aetiology, papillary muscle rupture is a life-threatening condition. The key diagnostic tool is echocardiography, and surgery should not be delayed.

## Supplementary Material

ytaf337_Supplementary_Data

## Data Availability

The data underlying this article are available in the article and its online Supplementary material.

## Supplementary material

**Consent:** The patient consented to the publication of this case report in compliance with the COPE guidelines.

**Funding:** None declared.
